# Prediction of Mortality after Burn Surgery in Critically Ill Burn Patients Using Machine Learning Models

**DOI:** 10.3390/jpm12081293

**Published:** 2022-08-06

**Authors:** Ji Hyun Park, Yongwon Cho, Donghyeok Shin, Seong-Soo Choi

**Affiliations:** 1 Department of Anesthesiology and Pain Medicine, National Medical Center, Seoul 04564, Korea; 2 Department of Radiology, Korea University Anam Hospital, University of Korea College of Medicine, Seoul 02841, Korea; 3 Department of Anesthesiology and Pain Medicine, Asan Medical Center, University of Ulsan College of Medicine, Seoul 05505, Korea

**Keywords:** burn, mortality, machine learning

## Abstract

Severe burns may lead to a series of pathophysiological processes that result in death. Machine learning models that demonstrate prognostic performance can be used to build analytical models to predict postoperative mortality. This study aimed to identify machine learning models with the best diagnostic performance for predicting mortality in critically ill burn patients after burn surgery, and then compare them. Clinically important features for predicting mortality in patients after burn surgery were selected using a random forest (RF) regressor. The area under the receiver operating characteristic curve (AUC) and classifier accuracy were evaluated to compare the predictive accuracy of different machine learning algorithms, including RF, adaptive boosting, decision tree, linear support vector machine, and logistic regression. A total of 731 patients met the inclusion and exclusion criteria. The 90-day mortality of the critically ill burn patients after burn surgery was 27.1% (198/731). RF showed the highest AUC (0.922, 95% confidence interval = 0.902–0.942) among the models, with sensitivity and specificity of 66.2% and 93.8%, respectively. The most significant predictors for mortality after burn surgery as per machine learning models were total body surface area burned, red cell distribution width, and age. The RF algorithm showed the best performance for predicting mortality.

## 1. Introduction

Burns are one of the most devastating types of traumatic injuries, causing morbidity and mortality worldwide. Burn wounds induce an excessive inflammatory response that triggers the immune system to protect against risk of infection, which can be harmful and potentially fatal [1]. The inflammatory mediators produced and released after a burn injury affect microcirculation, resulting in significant hypovolemic shock and substantial tissue injury [2]. The challenges of resuscitation and treatment with potential adverse outcomes have led to advances in the prediction of risk factors. Early detection and recognition of risk factors associated with mortality are essential in the management of a burn injury.

Machine learning is a type of artificial intelligence (AI) that leads to a superior prediction ability compared with conventional models, and has gained recent prominence [3]. Machine learning models have gained attention for their diagnostic performance, which can automatically build analytical models to predict postoperative mortality [4]. Recently, machine learning explainability is emphasized on decisions based on predictions provided by machine learning algorithms, which aid in making decisions to adopt the model [5]. Studies have reported the importance of explainable machine learning which can be applied to predict risk factors for mortality [6]. Mortality prediction is considered crucial in the early management of burn injuries, which can affect patient outcomes. Studies on machine learning models for mortality prediction in burn injuries have been in progress for decades [7,8]. The application of machine learning to burn injuries enables clinicians to reveal patterns and observe correlations that are not disclosed by traditional linear statistical analysis [9]. Efforts have been made to demonstrate the potential of machine learning approaches in predicting mortality in burn patients [10,11]. Not only for mortality prediction, machine learning is also being studied for the prediction of sepsis and acute kidney injury in burn patients, which are issues of concern [12]. However, the performance in predicting mortality after burn surgery using different machine learning techniques has not been clearly elucidated.

The aim of the study was to identify machine learning models with the best diagnostic performance for predicting mortality in patients after burn surgery and to compare each model’s suitability for this purpose. Our analysis used the following machine learning algorithms: random forest (RF), adaptive boosting (AB), decision tree (DT), support vector machine (SVM), and logistic regression (LGR). This study may help validate the use of machine learning models for applications in clinical practice.

## 2. Materials and Methods

### 2.1. Study Population

Critically ill burn patients admitted to the intensive care unit (ICU) before burn surgery from January 2010 to February 2018 were recruited. Patients with burns on more than 20% of their total body surface area (TBSA) are defined as critically ill burn patients [13]. Data immediately before the first burn surgery under general anesthesia were collected. The inclusion criteria were patients who underwent burn surgery within 14 days of a burn event, while patients under 18 years of age, those who underwent local anesthesia, and those with known chronic kidney disease were excluded from the study. We reviewed the electronic medical records of the patients to obtain laboratory and clinical data. This retrospective study was approved by the Institutional Review Board of the Ethical Committee of Hangang Sacred Heart Hospital, Hallym University, Seoul, Republic of Korea (No. 2018-057). The informed consent was waived by the committee due to the nature of retrospective design.

The primary outcome was the identification of risk factors for 90-day mortality after burn surgery using machine learning. The secondary outcome was the selection of the machine learning model with the best prediction performance.

### 2.2. Data Collection

Demographic data, laboratory data, and other patient variables were reviewed and collected using the electronic medical records system. Preoperative characteristics of the patients included sex, age, body mass index, history of hypertension or diabetes, American Society of Anesthesiologists physical status (ASA PS), TBSA burned, and the presence of inhalation injury. “TBSA burned” included a certain percentage of the body surface with a second- or third-degree burn. The presence of inhalation injury was diagnosed by bronchoscopic findings, with any findings other than normal considered an indication of the presence of inhalation injury. All preoperative blood tests were performed early in the morning of the day of surgery or the day before surgery. These preoperative laboratory data include hemoglobin, platelet count, prothrombin time (PT), albumin, creatinine, red cell distribution width (RDW), neutrophil–lymphocyte ratio (NLR), platelet–lymphocyte ratio (PLR), monocyte–lymphocyte ratio (MLR), and systemic immune-inflammation index (SII). NLR, PLR, MLR, and SII were each calculated using complete blood count (CBC) information. SII was calculated using the following formula: (granulocyte × platelet)/lymphocyte.

### 2.3. Primary Analysis of the Dataset

The baseline characteristics and laboratory findings were compared in the survivor and non-survivor groups 90 days after burn surgery. Risk factors for 90-day mortality after burn surgery were also identified using univariate and multivariate logistic regression analysis. The significant factors in univariate logistic regression were analyzed using the backward stepwise elimination procedure of multivariate logistic regression analysis. A two-tailed *p*-value < 0.05 was considered statistically significant. All statistical analyses were performed using SPSS for Windows (version 24.0; IBM-SPSS Inc., Armonk, NY, USA).

### 2.4. Clinical Feature Selection and Classification Method Using Machine Learning

**Figure 1:**
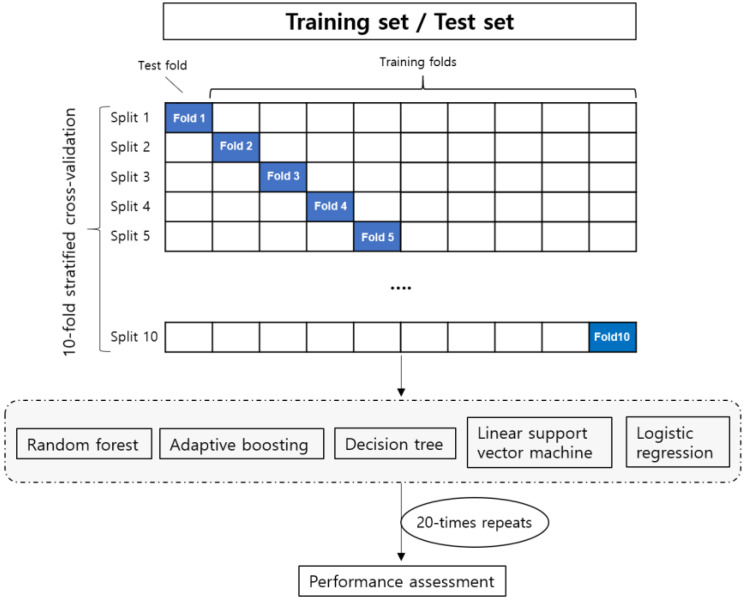
Schematic representation of 20 repeated 10-fold stratified cross-validations on training and test sets.

Although many quantitative features can be extracted from medical datasets, they may be highly correlated with each other, or simply noise. Thus, it is important to select a subset of features to enhance the performance and minimize the computational cost. Feature selection using RF regressor and 20 repeated 10-fold stratified cross-validations were performed to avoid overfitting in limited datasets (Figure 1) [14]. Important clinical features for predicting mortality in patients after burn surgery were selected using a RF regressor in Python (Python Software Foundation, version 3.7.4, Fredericksburg, VA, USA) with the Scikit-learn package [https://github.com/scikit-learn/scikit-learn (accessed on 25 September 2021)) [15]. A RF classifier model was trained to use the selected features to predict mortality [16]. The receiver operating characteristic (ROC) curve and classifier accuracy were used to compare the predictive accuracy of the RF, AB, DT, SVM, and LGR algorithms. Statistical differences in the AUC of each classifier were compared using a machine learning model with DeLong’s test using R (version 3.5.1; R Foundation for Statistical Computing, Vienna, Austria), with *p*-values < 0.05 considered statistically significant.

### 2.5. Algorithms of Each Machine Learning Model

The RF algorithm is an ensemble of many decision trees, which are non-linear models on various sub-samples of the dataset and calculate averaging to improve the predictive accuracy and prevent overfitting [16]. The importance of each feature is computed from the RF package of Scikit-learn. The RF algorithm is also known as the Gini importance.

- $G\left(N_{j}\right)=\overset{K}{\underset{i=1}{\sum }}p_{i}\left(1-p_{i}\right)=1-\overset{K}{\underset{i=1}{\sum }}p_{i}^{2}$

where *K* is the number of classes, and *P* is the probability of each class.

The AB classifier is a meta estimator that first fits a classifier on the original dataset, and then fits additional copies of the classifier on the same dataset with the weights of incorrectly classified instances adjusted, such that the subsequent classifiers focus more on difficult cases [17]. The AB classifier is also calculated using the following equation:

- $h_{f}\left(x\right)=\{\begin{array}{c} 1,\;if\;\overset{T}{\underset{t=1}{\sum }}(\log1/\beta_{t})h_{t}\left(x\right)\geq \;\frac{1}{2}\;\overset{T}{\underset{t=1}{\sum }}\log\frac{1}{\beta_{t}}\\ 0,\;otherwise \end{array}$

DT is a non-parametric supervised learning method for classification and regression. The goal of this method is to generate a model predicting a target value by learning simple decision rules inferred from the data features [18]. A tree can be seen as a piecewise constant approximation. For a classification outcome with values 0, 1, …, *K* − 1, for node,

- Pmk=1Nm∑y∈QmI(y=k)

is the proportion of class *k* observations in node *m*. If *m* is a terminal node, the predicted probability for this region is set to $P_{mk}$.

SVM constructs hyperplanes in a high or infinite dimensional space, which can be used for classification, regression, or other tasks. Intuitively, a good separation is achieved by a hyperplane that has the maximum gap to the nearest training data points of any class, because typically, the larger the margin, the lower the generalization error of the classifier [19,20]. The primal problem can be equivalently formulated as:

- $LinearSVR=min_{w,b}\frac{1}{2}w^{T}w+C\underset{i=1}{\sum }\max(0,\;|\;y_{i}-\left(w^{T}\emptyset \left(x_{i}\right)+b\right)|-\varepsilon ,$

where we make use of epsilon-insensitive loss, i.e., errors of less than ε are ignored. This is the form that is directly optimized by linear support vector regression (SVR).

LGR is a linear model for classification rather than regression. It quantifies the relationship between a dependent categorical outcome and one or more independent predictor variables. This implementation can fit binary, One-vs.-Rest, or multinomial logistic regression with optional $l_{1},\;l_{2}$ [21,22]. As an optimization problem, binary class $l_{1}$ penalized logistic regression minimizes the following cost function:

- $min_{w,c}\frac{1}{2}w^{T}w+C\overset{n}{\underset{i=1}{\sum }}\log\left(\exp\left(-y_{i}\left(X_{i}^{T}w+c\right)\right)+1\right)$

Similarly, $l_{2}$ regularized logistic regression solves the following optimization problem:

- $min_{w,c}{\left\|w\right\|}_{1}+C\overset{n}{\underset{i=1}{\sum }}\log\left(\exp\left(-y_{i}\left(X_{i}^{T}w+c\right)\right)+1\right)$

## 3. Results

### 3.1. Basic Demographics and Selected Important Clinical Features

Of 731 patients, there were 533 survivors and 198 non-survivors. Table 1 shows the baseline characteristics of the two groups. Univariate logistic regression analysis identified that age, diabetes, hypertension, ASA PS III and IV, TBSA burned, inhalation injury, RDW, platelet count, PT, albumin, and creatinine were significantly correlated with 90-day mortality of patients after burn surgery under general anesthesia (Table 2). Of these factors, age (odds ratio (OR) = 1.067, 95% confidence interval (CI) = 1.047–1.088, *p* < 0.001), diabetes (OR = 3.211, 95% CI = 1.288–8.000, *p* = 0.012), ASA PS III and IV (OR = 4.918, 95% CI = 1.581–15.305, *p* = 0.006), TBSA burned (OR = 1.095, 95% CI = 1.078–1.113, *p* < 0.001), RDW (OR = 1.679, 95% CI = 1.378–2.046, *p* < 0.001), PT (OR = 4.649, 95% CI = 1.259–17.171, *p* = 0.021), and creatinine (OR = 1.818, 95% CI = 1.181–2.798, *p* = 0.007) were considered independent risk factors in the multivariate logistic regression analysis. 

**Figure 2:**
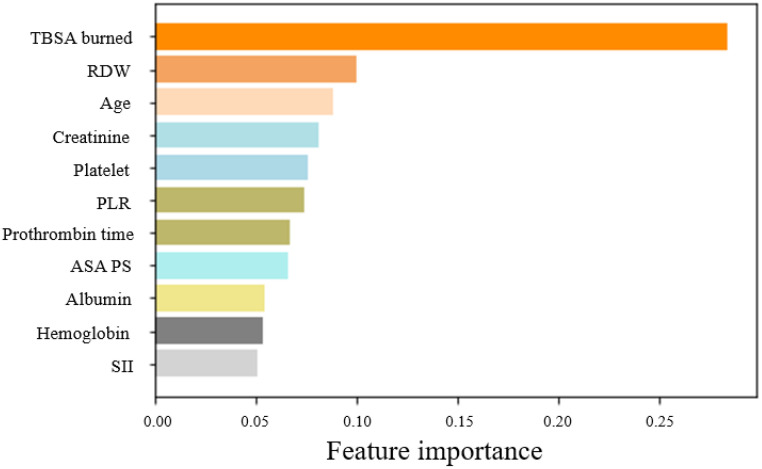
Plot of feature importance using random forest regressor. This figure shows the importance of each covariate in the final model. TBSA burned, RDW, and age achieved the highest feature importance in the machine learning models. TBSA: total body surface area; RDW: red cell distribution width; ASA PS, American Society of Anesthesiologists physical status; PLR, platelet to lymphocyte ratio; and SII, systematic immune-inflammation index.

The mean ages in the training and test datasets were 53.96 and 58.86, respectively. A total of 11 features were selected using the RF regressor: age, ASA PS, TBSA burned, hemoglobin, RDW, platelet count, PT, albumin, creatinine, PLR, and SII. Of these 11 features, the most significant predictors were TBSA (0.28447 ± 0.28447), RDW (0.10053 ± 0.10053), and age (0.08842 ± 0.08842) (Table 3). This is depicted as a histogram in Figure 2.

### 3.2. Diagnostic Performance of Each Machine Learning Model

**Figure 3:**
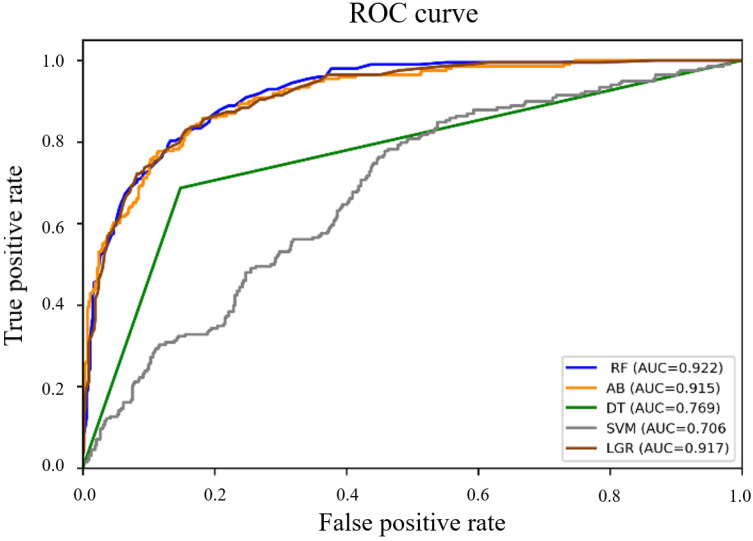
ROC curve comparing AUCs of different machine learning models and logistic regression model. ROC: receiver operating characteristic; RF: random forest; AB: adaptive boosting; DT: decision tree; SVM: support vector machine; LGR: logistic regression.

Figure 3 compares the AUCs of different machine learning models. RF achieved the highest AUC (0.922, 95% CI 0.902–0.942), with sensitivity and specificity of 66.2 and 93.8%, respectively. The AUCs of AB, DT, SVM, and LGR were 0.915 (95% CI 0.883–0.947), 0.769 (95% CI 0.705–0.833), 0.706 (95% CI, 0.582–0.829), and 0.917 (95% CI 0.895–0.939), respectively. Table 4 shows the AUC, sensitivity, specificity, positive predictive value (PPV), and negative predictive value (NPV) of each machine learning model.

### 3.3. Pairwise Comparison of AUC among Machine Learning Models

**Figure 4:**
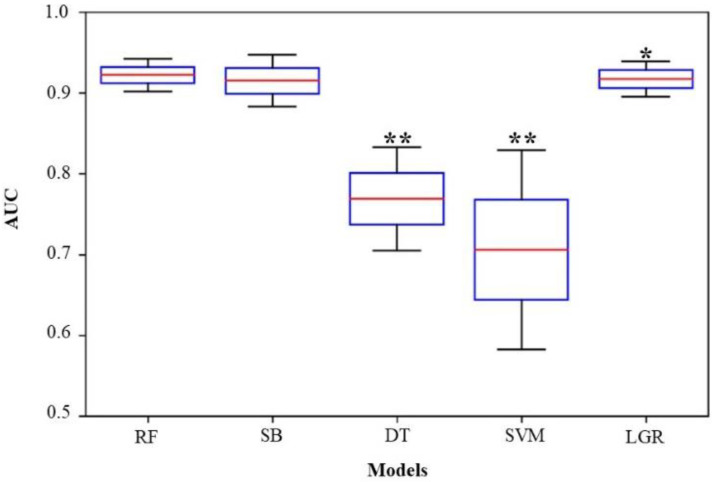
Box-and-whisker plot of the area under the AUC using DeLong’s test. RF showed no statistical difference with AB (*p* = 0.359). However, comparisons of RF with DT and SVM showed a significant difference, with *p* < 0.001 (**). Comparisons between RF and LGR also showed significant difference, with *p* < 0.05 (*). RF: random forest; AB: adaptive boosting; DT: decision tree; SVM: support vector machine; LGR: logistic regression; AUC: area under the receiver operating characteristic curve.

The pairwise comparisons of the AUCs using DeLong’s test demonstrated that RF (AUC = 0.922) showed no statistical difference with AB (AUC = 0.915) (*p* = 0.359) (Figure 4). However, comparisons between RF and DT (AUC = 0.769), SVM (AUC = 0.706), or LGR (AUC = 0.917) showed a significant difference (*p* < 0.05).

## 4. Discussion

In this study, we applied a machine learning approach to clinical features to compare models that predict patient mortality after burn surgery. RF achieved the highest AUC (0.922) among the evaluated models. Additionally, the pairwise comparisons of AUCs demonstrated that RF showed no statistical difference with AB. However, comparisons between RF and DT, SVM, and LGR showed a significant difference.

Using machine learning, the current study identified the most significant predictors of mortality after burn surgery as TBSA burned, RDW, and age. Among the 11 clinical features analyzed, TBSA burned constituted almost 30% of the feature importance. The feature importance of the other clinical features in descending order is RDW, age, creatinine, platelet count, PLR, prothrombin time, ASA PS, albumin, hemoglobin, and SII, with each forming less than 10% of the importance. TBSA burned is well known for its strong association with mortality in burn patients [23]. Additionally, RDW and age showed high feature importance among the clinical features. Clinical laboratory results such as creatinine, platelet count, PT, and PLR are significant risk factors in burn patients. This result is consistent with previous studies using classic logistic regression analysis [24,25].

The extent of injury is described using the percentage of the TBSA affected by a burn. The evaluation of TBSA burned is important for the initial burn management to estimate fluid requirements. TBSA burned is known to be a risk factor of mortality in burn injury, because higher TBSA leads to a poor prognosis [26]. Age is another well-known risk factor of mortality in burn patients. The underlying medical conditions, impaired response to infection, decreased ability to tolerate stress and physiological insult, and poor nutritional status associated with old age may cause adverse outcomes in elderly patients after burn injury [27,28].

Several preoperative laboratory variables have been analyzed for their predictive ability of mortality in burn patients. CBC is a routinely applied laboratory blood test for most patients. The unique components analyzed by CBC are known to be related with inflammation or infection that affects the prognosis of medical conditions [24]. Of these simple blood biomarkers, RDW is a numerical measurement of the range in the volume and size of the erythrocytes. An increase in RDW may reflect conditions that modify erythrocyte shapes as a result of premature release of immature cells into the bloodstream, as in the case of massive blood loss [29]. In addition, reports have shown that inflammation contributes to an increased RDW by inhibiting the production of erythropoietin or by decreasing erythrocyte survival [30,31]. Recently, RDW’s prognostic ability to predict morbidity and mortality in various clinical conditions has been demonstrated [32]. In burn patients, high RDW has been associated with adverse outcomes with mortality, but not as an independent risk factor [33,34]. However, in this study, we found that preoperative RDW is an independent predictive factor for 90-day mortality in patients after burn surgery using multivariate regression, as well as in the evaluations using machine learning.

Machine learning is a subset of AI that develops algorithms and technologies that enable computers to learn. Machine learning is a statistical method for extracting regularities from data. Machine learning uses various models or algorithms to extract data, predict, and classify their laws. Application of machine learning has advanced recently in various aspects of medicine [3]. Logistic regression is a traditional model commonly employed in medical applications to interpret clinical data in depth. Recent machine learning models include RF, AB, DT, SVM, and LGR, which are methods used to find a more optimal predictive model [35,36].

Among these machine learning models, our study demonstrated that RF showed the best performance in terms of predicting mortality in patients after burn surgery. Additionally, RF was not significantly different from AB. Despite the high AUC values of RF and AB, PPV and NPV were not high. Thus, the selection of the appropriate machine learning model to be used in clinical situations depends on the user.

Machine learning approaches have recently been reported to have better predictive abilities than classic statistical analysis. Regarding machine learning techniques in burn research, burn injury and management can be recognized as patterns that can capture non-linearities shown in independent features such as TBSA burned, age, or inhalation injury, which is different from conventional statistical approaches [37]. Another study about predicting mortality of burn patients was conducted using artificial neural networks, which included 15 clinical features, including inhalation injury, TBSA burned, and admission period [38]. To our knowledge, the current study is the first attempt at evaluating the clinical features of the patients to assess 90-day mortality after burn surgery using machine learning, with AUC as the performance metric.

The conducted analysis had some limitations. First, since this is a single-center study; institutional characteristics may have contributed to the survival of the burn patients. Perioperative clinical management might have changed over the eight-year period over which the patient data were collected. Thus, the results cannot be generally applied to burn patients. However, since the data used in this study were collected in the largest burn center in Asia, which performs standardized burn surgery, the effects on the present outcome may be minimal. Second, there was data loss or inaccurate data due to the retrospective design, which resulted in a relatively small dataset. Third, the models use the baseline preoperative characteristics without postoperative data. Although a dynamic model with sequential data may be superior, the model in our study predicts mortality within a specific period, which may be significant. Fourth, this study did not include the machine learning explainability techniques which may have provided a better understanding of how the models yield to their predictions. Finally, the additional data not included in our clinical features may have improved prediction. Further prospective studies are needed concerning these additional data with common clinical features for clinical acceptance.

## 5. Conclusions

This study demonstrated that the most significant predictors for 90-day mortality after burn surgery are percentage of burned TBSA, RDW, and age, using machine learning techniques. The RF algorithm showed the best performance for predicting mortality among the machine learning models evaluated. Pairwise comparisons demonstrated that RF showed no statistical difference with AB. However, comparisons between RF and DT, SVM, or LGR showed a significant difference. Further investigation in the future on a larger cohort with composite factors may help support the validity of the machine learning models.

## Figures and Tables

**Table 1:** Baseline characteristics and laboratory findings of the survivor and non-survivor groups 90 days after burn surgery.

Variable	Survivor Group (*n* = 533)	Non-Survivor Group (*n* = 198)	*p*-Value
Age, years	52.0 ± 14.4	58.0 ± 15.9	<0.001
Sex, male/female	441 (82.7)/92 (17.3)	166 (83.8)/32 (16.2)	0.825
Body mass index, kg/m^2^	23.6 ± 3.4	23.5 ± 3.1	0.765
Diabetes	22 (4.1)	26 (13.1)	<0.001
Hypertension	72 (13.5)	45 (22.7)	0.003
ASA PS			<0.001
I/II/III and IV	71 (13.3)/240 (45.0)/222 (41.7)	6 (3.0)/26 (13.1)/166 (83.8)	
TBSA burned, %	38.5 ± 15.1	63.6 ± 20.7	<0.001
Inhalation injury	165 (31.0)	110 (55.6)	<0.001
Hemoglobin, g/dL	13.5 ± 3.0	13.9 ± 3.5	0.100
RDW	13.0 ± 1.0	13.8 ± 1.4	<0.001
Platelet count, ×10^9^/L	204.8 ± 111.3	180.5 ± 133.8	0.023
Prothrombin time, INR	1.1 ± 0.2	1.2 ± 0.3	<0.001
Albumin, g/dL	2.9 ± 0.8	2.5 ± 0.9	<0.001
Creatinine, mg/dL	0.78 ± 0.42	1.02 ± 0.62	<0.001
NLR	10.6 ± 19.1	11.2 ± 15.7	0.695
PLR	276 ± 464	304 ± 606	0.553
MLR	0.85 ± 1.31	1.13 ± 2.39	0.121
SII	2171 ± 4108	1909 ± 3783	0.435

Data are shown as mean ± standard deviation or number (%) as appropriate. ASA PS: American Society of Anesthesiologists physical status; INR: international normalized ratio; MLR: monocyte–lymphocyte ratio; NLR: neutrophil–lymphocyte ratio; PLR: platelet–lymphocyte ratio; RDW: red cell distribution width; SII: systemic immune-inflammation index; TBSA: total body surface area.

**Table 2:** Univariate and multivariate analyses for evaluating the risk factors of mortality after burn surgery.

	Univariate Analysis		Multivariate Analysis	
Variables	Odds Ratio (95% CI)	*p*-Value	Odds Ratio (95% CI)	*p*-Value
Age, years	1.027 (1.016–1.039)	<0.001	1.067 (1.047–1.088)	<0.001
Diabetes mellitus	3.511 (1.940–6.356)	<0.001	3.211 (1.288–8.000)	0.012
Hypertension	1.883 (1.244–2.852)	0.003	1.348 (0.683–2.660)	0.389
ASA PS				
I	1.000 (Reference)		1.000 (Reference)	
II	1.282 (0.508–3.237)	0.599	1.101 (0.329–3.681)	0.876
III and IV	8.848 (3.755–20.852)	<0.001	4.918 (1.581–15.305)	0.006
TBSA burned, %	1.075 (1.063–1.087)	<0.001	1.095 (1.078–1.113)	<0.001
Inhalation injury	2.788 (1.994–3.898)	<0.001	1.380 (0.844–2.257)	0.199
Hemoglobin, g/dL	1.048 (0.995–1.104)	0.075		
RDW	1.711 (1.471–1.990)	<0.001	1.679 (1.378–2.046)	<0.001
Platelet count, ×10^9^/L	0.998 (0.997–1.000)	0.014	0.999 (0.997–1.001)	0.477
Prothrombin time, INR	29.531 (10.480–83.213)	<0.001	4.649 (1.259–17.171)	0.021
Albumin, g/dL	0.596 (0.480–0.741)	<0.001	0.981 (0.686–1.404)	0.916
Creatinine, mg/dL	2.894 (1.908–4.391)	<0.001	1.818 (1.181–2.798)	0.007
NLR	1.002 (0.993–1.010)	0.696		
PLR	1.000 (1.000–1.000)	0.506		
MLR	1.090 (0.994–1.195)	0.068		
SII	1.000 (1.000–1.000)	0.440		

CI, confidence interval; ASA PS, American Society of Anesthesiologists physical status; TBSA, total body surface area; RDW, red cell distribution width; NLR, neutrophil–lymphocyte ratio; PLR, platelet–lymphocyte ratio; MLR, monocyte–lymphocyte ratio; SII, systemic immune-inflammation index; INR, international normalized ratio.

**Table 3:** Feature importance of the variables associated with mortality after burn surgery.

Variables	Feature Importance
TBSA burned	0.28447 ± 0.28447
RDW	0.10053 ± 0.10053
Age	0.08842 ± 0.08842
Creatinine	0.08194 ± 0.08194
Platelet	0.07586 ± 0.07586
PLR	0.07459 ± 0.07459
Prothrombin time	0.06747 ± 0.06747
ASA PS	0.06676 ± 0.06676
Albumin	0.05457 ± 0.05457
Hemoglobin	0.05401 ± 0.05401
SII	0.05139 ± 0.05139

TBSA—total body surface area; RDW—red cell distribution width; ASA PS—American Society of Anesthesiologists physical status; PLR—platelet to lymphocyte ratio; and SII—systematic immune-inflammation index.

**Table 4:** AUC, sensitivity, specificity, PPV, and NPV of each machine learning model.

Model	AUC (95% CI)	Sensitivity	Specificity	PPV	NPV
RF	0.922 (0.902–0.942)	66.2%	93.8%	79.9%	88.2%
AB	0.915 (0.883–0.947)	69.2%	91.2%	74.5%	88.8%
DT	0.769 (0.705–0.833)	68.7%	85.2%	63.3%	88.0%
SVM	0.706 (0.582–0.829)	3.0%	99.0%	54.5%	73.3%
LGR	0.917 (0.895–0.939)	68.7%	92.7%	77.7%	88.8%

RF—random forest; AB—adaptive boosting; DT—decision tree; SVM—support vector machine; LGR—logistic regression; AUC—area under the receiver operating characteristic curve; PPV—positive predictive value; and NPV—negative predictive value.

## Data Availability

Not applicable.
